# Association Between Day‐to‐Day Variation in Electroencephalographic (EEG) Sleep Features and Daytime Cognitive Function in Adults at Risk for Dementia

**DOI:** 10.1002/alz70860_107491

**Published:** 2025-12-23

**Authors:** Dharmendra Gurve, Andrew Peter Centen, Penelope J. Slack, Thien Thanh Dang‐Vu, Sylvie Belleville, Nicole D. Anderson, Manuel Montero‐Odasso, Haakon B. Nygaard, Howard Chertkow, Howard H. Feldman, Paul W.H. Brewster, Andrew Lim, for the CCNA‐CAN‐THUMBS‐UP Study Group

**Affiliations:** ^1^ University of Toronto, Toronto, ON, Canada; ^2^ Sunnybrook Health Sciences Centre, Toronto, ON, Canada; ^3^ University of British Columbia, Vancouver, BC, Canada; ^4^ Centre de recherche de l'Institut universitaire de gériatrie de Montréal, Montreal, QC, Canada; ^5^ PERFORM Centre, Concordia University, Montreal, QC, Canada; ^6^ Concordia University, Montreal, QC, Canada; ^7^ Research Centre, Institut universitaire de gériatrie de Montréal, Montreal, QC, Canada; ^8^ Department of Psychology, Université de Montréal, Montreal, QC, Canada; ^9^ Rotman Research Institute, Baycrest Academy for Research and Education, Toronto, ON, Canada; ^10^ Rotman Research Institute, Toronto, ON, Canada; ^11^ Ben & Hilda Katz Interprofessional Research Centre in Geriatric and Dementia Care, Toronto, ON, Canada; ^12^ Kimel Family Centre for Brain Health and Wellness, Toronto, ON, Canada; ^13^ Western Univeristy, London, ON, Canada; ^14^ Lawson Health Research Institute, London, ON, Canada; ^15^ Schulich School of Medicine & Dentistry, Division of Geriatric Medicine, Western University, London, ON, Canada; ^16^ UBC Hospital Clinic for Alzheimer Disease and Related Disorders, Vancouver, BC, Canada; ^17^ Division of Neurology, Department of Medicine, University of Toronto, Toronto, ON, Canada; ^18^ Kimel Family Centre for Brain Health and Wellness and Anne & Allan Bank Centre for Clinical Research Trials, Baycrest Health Sciences, Toronto, ON, Canada; ^19^ Baycrest and Rotman Research Institute, Toronto, ON, Canada; ^20^ Baycrest Academy, Toronto, ON, Canada; ^21^ University of California San Diego, Department of Neurosciences, La Jolla, CA, USA; ^22^ Alzheimer's Disease Cooperative Study (ADCS), University of California San Diego, La Jolla, CA, USA; ^23^ Cognition & Technology Research Group, Institute on Aging and Lifelong Health, University of Victoria, Victoria, BC, Canada; ^24^ University of Victoria, Victoria, BC, Canada

## Abstract

**Background:**

Older adults experience considerable day‐to‐day variability in cognitive function. We aimed to test the hypothesis that this is in part related to sleep, and determine which EEG sleep features are most important in supporting day to day cognitive resilience.

**Method:**

We analyzed data from 149 adults at high risk for dementia participating in the Brain Health Pro (BHPro) study. At BHPro baseline, participants underwent up to 3 nights of overnight ambulatory EEG using the MUSE‐S (Interaxon, Toronto, Canada) as well as multi‐day app‐based cognitive testing (MyCogHealth, Victoria, Canada). Of 350 participants, 149 had EEG and cognitive evaluation that overlapped by at least 1 day. We performed automated sleep staging and computed frontal NREM (N2 and N3) delta power and REM theta power. We used linear mixed effect models to relate each morning's composite global cognitive test results to the previous night's sleep measures.

**Result:**

149 individuals had >=1 cognitive evaluation within 12 hours of an overnight EEG recording. Of these, 63 had 2 nights, and 37 had >=3 nights. Greater % REM sleep (+0.15 per 1SD greater REM sleep, SE 0.04 *p* = 0.0001) and relative REM theta power (+0.08 per 1SD greater relative REM theta power, SE 0.04, *p* = 0.02) the night before were associated with better cognitive performance the next morning, and there was a non‐significant positive relationship (+0.06 per 1SD difference, SE 0.04, *p* = 0.11) between NREM delta power and cognitive performance the following morning. These effects were particularly strong in those with mild cognitive impairment (delta power interaction *p* = 0.055; theta power interaction *p* = 0.02)

**Conclusion:**

REM sleep theta power and NREM delta power may support day to day cognitive performance in older adults at high risk for dementia, particularly those with mild cognitive impairment, and may represent electrophysiologic therapeutic targets to support cognitive resilience.

